# Differential attraction of drosophilids to banana baits inoculated with *Saccharomyces cerevisiae* and *Hanseniaspora uvarum* within a Neotropical forest remnant

**DOI:** 10.7717/peerj.3063

**Published:** 2017-03-09

**Authors:** Marcos R.D. Batista, Fabiana Uno, Rafael D. Chaves, Rosana Tidon, Carlos A. Rosa, Louis B. Klaczko

**Affiliations:** 1 Depto. de Genética, Evolução e Bioagentes, Inst. de Biologia, Universidade Estadual de Campinas – UNICAMP, Campinas, São Paulo, Brazil; 2 Depto. Ciência de Alimentos, Fac. Engenharia de Alimentos, Universidade Estadual de Campinas – UNICAMP, Campinas, São Paulo, Brazil; 3 Depto. Genética e Morfologia, Inst. Ciências Biológicas, Universidade de Brasília – UnB, Brasília, DF, Brazil; 4 Depto. Microbiologia, ICB, Universidade Federal de Minas Gerais – UFMG, Belo Horizonte, Minas Gerais, Brazil

**Keywords:** Food preference, Yeast, *Drosophila*, *Drosophila* assemblage, Atlantic Rainforest, *Tripunctata*, *Willistoni*, *Melanogaster*, Exotic *drosophila*, *Guarani*

## Abstract

**Background:**

Yeasts are a necessary requisite in the diet of most *Drosophila* species that, in turn, may vector their dispersal in natural environments. Differential attractiveness experiments and the isolation of yeasts consumed by *Drosophila* may be informative for characterizing this association. *Hanseniaspora uvarum* is among the most common yeast species isolated from *Drosophila* crops, with high attractiveness to drosophilids. *Saccharomyces cerevisiae* has been widely used to collect flies, and it allows broad sampling of almost all local *Drosophila* species. Pronounced differences in the field concerning *Drosophila* attractivity to baits seeded with these yeast species have been previously reported. However, few explicit generalizations have been set. Since late fifties, no field experiments of *Drosophila* attractivity were carried out in the Neotropical region, which is facing shifts in abiotic and biotic factors. Our objective is to characterize preference behavior that mediates the interaction in the wild among Neotropical *Drosophila* species and yeasts associated with them. We want to set a broad generalization about drosophilids attracted to these yeasts. Here we present the results of a differential attractiveness experiment we carried out in a natural Atlantic Rainforest fragment to assess the preferences of *Drosophila* species groups to baits inoculated with *H. uvarum* and *S. cerevisiae*.

**Methods:**

Both yeast species were cultured in GYMP broth and separately poured in autoclaved mashed banana that was left fermenting. In the field, we collected drosophilids over five arrays of three different baits: non-inoculated autoclaved banana and banana inoculated with each yeast. In the laboratory the drosophilids were sorted to five sets according to their external morphology and/or genitalia: *tripunctata*; *guarani*; *willistoni*; *exotic*; and the remaining flies pooled in *others*.

**Results and Conclusions:**

Uninoculated banana baits attracted virtually no flies. We found significant departures from random distribution over the other two baits (1:1 proportion) for all sets, except the pooled *others*. Flies of the sets *willistoni* and *exotic* preferred *H*. *uvarum* over *S*. *cerevisiae,* while the remaining sets were more attracted to *S*. *cerevisiae*. Previously, various authors reported similar patterns in attraction experiments with *S*. *cerevisiae* and *H*. *uvarum*. It is also noteworthy that both yeast species have been isolated from natural substrates and crops of *Drosophila* species. Taken together, these results suggest that the preferences among *Drosophila* species groups may be reflecting deep and stable relations with yeast species in natural environments. They can be summarized as: forest dwelling species from subgenus *Drosophila* (such as *tripunctata* and *guarani* groups) are attracted to banana baits seeded with *S*. *cerevisiae*; while exotic (as *D. melanogaster*) and subgenus *Sophophora* species are preferentially attracted to baits seeded with *H*. *uvarum*.

## Introduction

*Drosophila*-yeast association can be considered a diffuse mutualism (Starmer & Lachance, 2011; Buser et al., 2014), in which yeasts are a necessary requisite in the diet of most *Drosophila* species (Powell, 1997) that, in turn, may vector their dispersal in natural environments (Ganter, 1988; Christiaens et al., 2014). Moreover, *Drosophila* adults and larvae regulate yeast composition and density in natural substrates (Stamps et al., 2012), while different yeast species affect *Drosophila* breeding (Barker, 1992) and feeding preferences (Becher et al., 2012) as well as bionomic features (Anagnostou, Dorsch & Rohlfs, 2010).

Traditionally, this association is characterized by isolating yeasts from *Drosophila* crops (Phaff et al., 1956) and natural substrates (Carson, Knapp & Phaff, 1956; Starmer, 1981; Barker, Starmer & Vacek, 1987); also, by investigating *Drosophila* species attraction to baits inoculated with different yeast species in the field (Da Cunha, Dobzhansky & Sokoloff, 1951; Klaczko, Powell & Taylor, 1983) and in the laboratory (Barker et al., 1981; Becher et al., 2012; Palanca et al., 2013). These papers show the essential role yeasts play for the attractiveness of fruit baits and fermenting substrates (see also: Walsh et al., 2011; Hamby et al., 2012; Kleiber et al., 2014).

*Hanseniaspora uvarum* (= *Kloeckera apiculata*) is among the most common yeast species isolated from *Drosophila* crops in different parts of the world (828/2222 yeast OTUs of 15 *Drosophila* populations reported by Chandler, Eisen & Kopp (2012)). Its prevalence is about 50% in association with species of *D. melanogaster* group in North America (78/163 reported by Camargo & Phaff (1957); and 173/344 by Chandler, Eisen & Kopp (2012)). In the Neotropical region, its prevalence associated with *D. willistoni* from the Amazon is close to 50% (85/174 isolates, see Morais et al., 1995) and almost 40% with *D.  willistoni* populations from the Atlantic Rainforest (146/394 isolates, see Da Cunha, Shehata & De Oliveira, 1957). Additionally, banana baits seeded with *H. uvarum* have been used since the early fifties in attractiveness experiments due to its easy growth on bananas and high attractiveness of *Drosophila* specimens (Da Cunha, Dobzhansky & Sokoloff, 1951).

Despite evidences of different substrates attracting distinct assortments of resident *Drosophila* (Dobzhansky & Pavan, 1950; Del Pino et al., 2015), mashed banana fermented with *Saccharomyces cerevisiae* has been used since the dawn of *Drosophila* research to collect and to raise flies (Loeb & Northrop, 1916; Dobzhansky, 1936; Reed, 1938; and others; see Spencer, 1950 for a review of the early use of banana baits seeded with baker’s yeast). This kind of baits allows a broad sampling with almost all resident *Drosophila* species (see Da Cunha, Dobzhansky & Sokoloff, 1951). Therefore, it has been viewed as a control treatment concerning bait attractiveness in the field or an all-purpose bait.

Previous studies have examined *Drosophila* attractivity to baits seeded with *H. uvarum* and *S. cerevisiae* in the field (Da Cunha, Dobzhansky & Sokoloff, 1951; Da Cunha, Shehata & De Oliveira, 1957; Klaczko, Powell & Taylor, 1983). Pronounced differences concerning the abundance of *Drosophila* species collected over baits with these yeasts are described. However, due to technical complexity in their experimental design (for example, the use of various baits with different yeast species simultaneously), few explicit generalizations could be set when comparing the attractiveness of these yeast species.

The biodiversity of *Drosophila* in the Neotropical region is rich (Val, Vilela & Marques, 1981), especially in the Atlantic Rainforest biome, where half of the species remains to be described (Medeiros & Klaczko, 2004). Furthermore, phylogenetic (Yotoko et al., 2003; Hatadani et al., 2009; Izumitani et al., 2016) and morphological (Throckmorton, 1975) differences among *Drosophila* species groups are so great that they may be considered—and have been used as—a valid taxonomic classification for characterizing patterns of abundance and distribution in especially rich environments (Dobzhansky & Pavan, 1950; Dobzhansky & Da Cunha, 1955).

Since the late 1950s (Da Cunha, Shehata & De Oliveira, 1957), no experiments examining *Drosophila* differential attractivity in the Neotropical region were carried out. Moreover, due to climate change (Lemes, Melo & Loyola, 2014) and forest fragmentation (Ribeiro et al., 2009) environmental conditions in the Atlantic Rainforest biome are becoming more heterogeneous, with pronounced shifts for local fauna (Batista, Ananina & Klaczko, 2012; Batista & Klaczko, 2013) and flora (Carvalho, Braga & Nascimento, 2016). Furthermore, new occurrence of invasive drosophilid species, such as *Zaprionus indianus* (see Vilela, 1999) and *D. suzukii* (see Deprá et al., 2014; Vilela & Mori, 2014), have probably affected ecological interactions among taxa from this biome.

Our objective is to characterize preference (breeding and feeding) behavior that mediates the interaction in the wild among Neotropical *Drosophila* species and yeasts naturally associated with them. Previous studies have repeatedly shown differences of *Drosophila* attractivity to baits seeded with *H. uvarum* and *S. cerevisiae.* However, so far no clear generalization has been made for the attractivity in the wild. Thus, as a first step in this endeavor, we want to assess the preferences of *Drosophila* species (groups), from a Neotropical forest fragment, to baits inoculated with either of two yeast species: *H. uvarum*, one of the yeast species most commonly associated with *Drosophila*; and *S. cerevisiae*, the most commonly used yeast species for collecting these flies. Our working hypothesis is that these two yeast species attract sets of flies with different proportions of *Drosophila* species.

## Material and Methods

The *Drosophila* attraction experiment was carried out within a forest fragment of the Atlantic Rainforest located at Itatiba, SP, Brazil (23°00.07′S, 46°52.917′W; altitude: 740 m) on October 22, 2014 (Permanent Field Permit for Collecting Zoological Material from IBAMA, ICMBio, Ministério do Meio Ambiente—MMA, number: 17238-1). This forest fragment is located 88 km northern Serra da Cantareira, SP, Brazil where Da Cunha, Shehata & De Oliveira (1957) carried out their experiments. Floristic and climatologic properties of both localities are similar, since they belong to the same orogenic formation—Serra da Mantiqueira (Ross, 2013).

We started our experiment around 06h30 a.m., when we randomly exposed baits in the field, and swept entomological nets over baits every 15 min until noon. Then, between 04h00 p.m. and 06h00 p.m., the same procedure was repeated. This strategy was adopted, to minimize possible effects of aggregation behavior and daily temperature variation.

Two different yeast species (commercial *S. cerevisiae* and *H. uvarum*—strain ACL-35D; deposited under code UFMG-CM-Y4001 in the Collection of Microorganisms and Cells of the Federal University of Minas Gerais, Brazil) were cultured for 48 h in 200 ml GYMP broth (2% glucose, 0.5% yeast extract, 1% malt extract and 0.2% sodium phosphate monobasic monohydrate). This procedure usually in our laboratory produces suspensions with concentration of 10^7^–10^8^ cells/ml. Then, 200 ml yeast suspensions were poured and stirred with a sterile spoon over approximately 1.44 kg of autoclaved mashed banana; which were left to ferment for about 20 h. On October 22, 2014, temperature varied between 16.5°C and 30.5°C, and the average daily temperature was 23.5°C (see https://www.agritempo.gov.br/agritempo/index.jsp?lang=en, meteorological station CEPAGRI—Campinas, SP). We used field proceedings similar to those described by Da Cunha, Shehata & De Oliveira (1957), when they used mashed banana seeded with *H. uvarum* and *S. cerevisiae* with positive yeast growth confirmed by the fermentation of the banana bait and a noticeable bouquet.

Three kinds of banana baits (non-inoculated autoclaved banana and autoclaved banana inoculated with each of the two yeast species) were randomly placed, in order to avoid bias related to position effects, at the edges of an equilateral triangle inscribed in a circle of about 3.5 m of diameter. Each set was distant 10 m from the next set. We collected over five sets of three baits with a total of 15 baits. We collected drosophilids over each type of baits separately, stored them in separate vials, and brought them alive to the laboratory to be analyzed.

Flies were sorted to five groups (see Table 1): *tripunctata* (*D. tripunctata* species group); *guarani* (*D. guarani* species group); *willistoni* (*D. willistoni* species group); *exotic* (*D. immigrans*, *D. melanogaster* species group and *Zaprionus indianus*); and the remaining flies pooled in *others* (*D. calloptera*, *D. cardini* species group and other non-identified drosophilids). We used *Drosophila* species group identification as proposed by Freire-Maia & Pavan (1949).

**Table 1:** Drosophilids collected over baits with *H. uvarum*, *S. cerevisiae* and without yeast. Number of females (♀) and males (♂) of drosophilid species collected over three kinds of baits: **control**—autoclaved banana without yeast; autoclaved banana with *H. uvarum*; autoclaved banana with *S. cerevisiae*; ∑— sum of females and males. Field trip held on October 22, 2014 at Itatiba, SP, Brazil (23°00.07′S, 46°52.917′W; altitude: 740 m).

Group	Control			*H. uvarum*			*S.cerevisiae*			Total
Species	♀	♂	∑	♀	♂	∑	♀	♂	∑	
***Tripunctata* group**										
*D. bandeirantorum*	0	0	0	0	3	3	0	1	1	**4**
*D. bifilum*	0	0	0	0	0	0	0	1	1	**1**
*D. cuaso*	1	1	2	0	0	0	0	1	1	**3**
*D. fragilis*	0	0	0	0	1	1	0	2	2	**3**
*D. mediopunctata*	1	2	3	2	2	4	4	13	17	**24**
*D. paraguayensis*	0	0	0	5	7	12	13	38	51	**63**
*D. paramediostriata*	0	0	0	0	1	1	0	0	0	**1**
*D. nappae*	0	0	0	0	0	0	0	3	3	**3**
*D. trifilum*	0	0	0	0	0	0	0	2	2	**2**
*Non-identified*	1	0	1	6	1	7	15	0	15	**23**
**Group total**			**6**			**28**			**93**	**127**
***Guarani*group**										
*D. griseolineata*	0	2	2	5	19	24	8	32	40	**66**
*D. maculifrons*	0	0	0	1	0	1	3	7	10	**11**
*Non-identified*	0	0	0	4	0	4	2	0	2	**6**
**Group total**			**2**			**29**			**52**	**83**
***Willistoni*group**										
*D. nebulosa*	0	0	0	11	2	13	2	0	2	**15**
*D. willistoni*	2	0	2	19	16	35	3	2	5	**42**
**Group total**			**2**			**48**			**7**	**57**
***Exotic*species**										
*D. immigrans*	0	0	0	3	1	4	0	0	0	**4**
*D. melanogaster*	0	0	0	0	1	1	0	0	0	**1**
*D. suzukii*	0	0	0	0	6	6	1	1	2	**8**
*D. simulans*	0	1	1	5	8	13	3	1	4	**18**
*Zaprionus indianus*	0	0	0	2	3	5	0	0	0	**5**
**Group total**			**1**			**29**			**6**	**36**
***Others***										
*D. atrata*	0	0	0	0	0	0	2	0	2	**2**
*D. polymorpha*	0	0	0	1	2	3	1	0	1	**4**
Drosophilids	0	0	0	4	0	4	0	0	0	**4**
**Group total**			**0**			**7**			**3**	**10**
**Total**			**11**			**141**			**161**	**313**

Wild male flies were identified to species level by dissecting their genitalia; and for collected females, the genitalia of their laboratory reared F1 males were analyzed. The specimen genitalia of *tripunctata* group flies was compared to drawings reported by: Frota-Pessoa (1954), Val (1982), Vilela & Pereira (1985), Vilela & Pereira (1986), Bächli, Vilela & Ratcov (2000) and Vilela, Valente & Basso-da-Silva (2004). Species of *calloptera*, *cardini* and *guarani* groups were compared to drawings reported by: Val (1982) and Vilela & Bächli (1990). Specimens that belong to *melanogaster* and *willistoni* groups were compared to drawings reported by: Salles (1948) and Malogolowkin (1952). We used external morphology for classification of *D. immigrans*, *D. suzukii* and *Z. indianus* specimens. When the genitalia were lost, flies were identified by external morphology and labeled as *non-identified* in the respective group.

Breeding and egg-laying preferences may motivate choice behavior of *Drosophila* females. Therefore, bias in sex ratio would be expected over a particular bait, if females would choose that substrate for oviposition. We performed a chi-square test for characterizing differences in sex ratio between baits. Since no bias in group sex ratio was found (see Table 2), we analyzed the sum of females and males collected over baits. After that, we compared the attractiveness of each set of baits testing the observed numbers of flies within each group collected over *S. cerevisiae* and *H. uvarum* against an expected 1:1 proportion with a chi-square.

**Table 2:** Comparisons between collected male and female proportions. Chi-square tests comparing the number of females (♀) and males (♂) in each *Drosophila* group collected over baits with ***H.*** ***uvarum*** and ***S.*** ***cerevisiae***.

Groups	*X*^2^	*d.f.*	*p*-value
*Tripunctata*	2.22	1	>0.1^ns^
*Guarani*	0.82	1	>0.3^ns^
*Willistoni*	0.21	1	>0.7^ns^
*Exotic*	2.15	1	>0.3^ns^

**Notes.**

*X*^2^ result of chi-square tests *d.f.* degree of freedom *p*-value associated probability ns non-significant

## Results

Table 1 shows the 313 specimens of drosophilids collected. Uninoculated banana baits (controls) attracted virtually no flies (11 versus 302 in the other ones; less than 4% of the total). Thus, the results of flies from uninoculated banana baits were no further analyzed.

**Figure 1:**
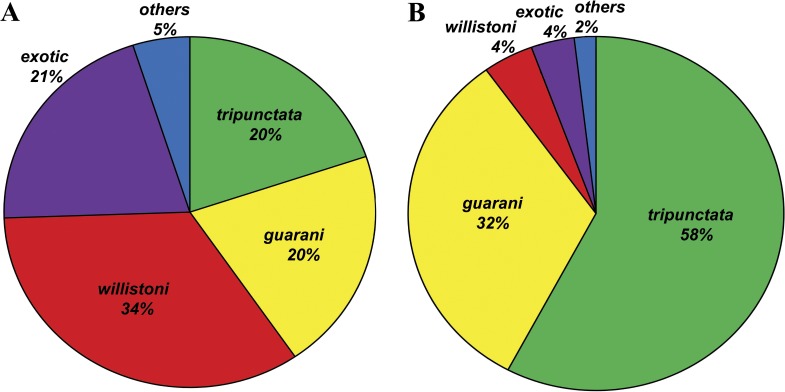
Drosophilids attracted to yeast-inoculated baits. Percentage of Drosophilidae groups (in green *tripunctata*; in yellow *guarani*; in red *willistoni*; in purple *exotic*; and in blue *others*) collected over baits inoculated with *Hanseniaspora uvarum* (A) and with *Saccharomyces cerevisiae* (B).

We collected 141 flies (47% of the 302 flies attacted to yeast inoculated baits) over baits with *H. uvarum* and 161 (53% of the total 302) over baits with *S. cerevisiae*. Species with largest numbers among the 20 species identified were *Drosophila griseolineata* (*n* = 66), *D. paraguayensis* (*n* = 63) and *D. willistoni* (*n* = 42). The *D. tripunctata* group was the most diverse with ten species, followed by *D. melanogaster* group with three species (pooled as *exotic* in Table 1). Although no significant difference was detected between total number of flies collected over baits seeded with the two diffent yeasts (*X*^2^ = 0.66; *d*.*f*. = 1; *p* > 0.30), the composition of flies was clearly different (Fig. 1).

Flies of the *tripunctata* and *guarani* groups (subgenus *Drosophila*) showed similar pattern and were significantly more attracted to baits inoculated with *S. cerevisiae* (93 in 121 = 77%, *X*^2^ = 34.9, *d*.*f*. = 1, *p* < 0.001; and 52 in 81 = 64%, *X*^2^ = 6.5, *p* < 0.01) than to *H. uvarum* (23% and 36%, respectively). However, flies of *willistoni* (7 in 55 = 13%; *X*^2^ = 30.6, *d*.*f*. = 1, *p* < 0.001), *exotic* (6 in 35 = 17%; *X*^2^ = 15.1, *p* < 0.01) and *other* (3 in 10 = 30%; *X*^2^ = 1.6, *p* > 0.2—*non-significant*) groups were less collected over *S. cerevisiae* than over *H. uvarum* (87%, 83%, and 70%, respectively). After Bonferroni multiple tests correction, all test significance results remain qualitatively unchanged.

## Discussion

Parts of plants or fungi with a particular microbiota are substrates used by several *Drosophila* species for feeding, mating, oviposition and breeding (Powell, 1997). Although fresh fruits are attractive for *D. suzukii* (see Keesey, Knaden & Hansson, 2015), most *Drosophila* species are attracted to decayed fruits. Furthermore, variation in microbiota density associated to its decaying age (in number of days) affects the attraction and abundance of several cosmopolitan species, such as *D. melanogaster*, *D. simulans* and *D. immigrans*, to decayed oranges (Nunney, 1996).

The low attractiveness of baits with non-fermented banana reinforces the fact of the presence of yeast are necessary for baits attractiveness (Klaczko, Powell & Taylor, 1983). Although no direct count of yeast colonies was carried out in the banana baits, the obvious banana fermentation (increased volume and typical bouquet) were compeling evidences of yeast growth. Additionally, we collected approximately the same magnitude of specimens over banana baits with each of the two yeasts, indicating both yeasts grew and the species composition differences could be attributed to differences in odor profiles between *H. uvarum* and *S. cerevisiae* (see Scheidler et al., 2015).

We collected more flies of subgenus *Sophophora* such as *D. melanogaster* and *D. suzukii* over baits with *H. uvarum* (68 in a total of 81 = 84%) than over *S. cerevisiae* (13/81 = 16%), while flies of the *tripunctata* group (subgenus *Drosophila*) were more attracted to baits inoculated with *S. cerevisiae* (93 in 121 = 77%) than to *H. uvarum* (23%).

Species that belong to subgenus *Sophophora*, such as *D. melanogaster* and flies of *D. obscura* group, showed preferences for baits inoculated with apiculate yeast *H. uvarum* over other yeasts, such as *S. cerevisiae* in laboratory populations (Hoang, Kopp & Chandler, 2015) and natural populations (Da Cunha, Dobzhansky & Sokoloff, 1951; Klaczko, Powell & Taylor, 1983). However, other species from subgenus *Drosophila*, such as *D. occidentalis* were more collected over baits with *S. cerevisiae* than over baits with apiculate yeasts in San Jacinto Mountains, CA, USA (Klaczko, Powell & Taylor, 1983).

In the tropical region, Dobzhansky & Da Cunha (1955) and Da Cunha, Shehata & De Oliveira (1957) carried out experiments of differential attractiveness in the Amazon (Belém, PA, Brazil and Tapajós, PA, Brazil) and in the Atlantic Rainforest (Rio Doce, MG, Brazil and Serra da Cantareira, SP, Brazil). These authors observed that flies of *Sophophora* subgenus, such as *D. willistoni*, *D. nebulosa*, and *D. simulans*, were more collected over baits with *H. uvarum* than over baits with *Candida krusei* (=*Pichia kudriavzevii*) and *S. cerevisiae* or close relatives. However, *H. uvarum* baits were poorly attractive to some species from subgenus *Drosophila* such as *D. calloptera, D. guaramunu* and flies from *D. tripunctata* group, which were collected over baits with yeast from genera *Candida*, *Pichia* and *Saccharomyces*.

Choice behavior may be triggered by females that are choosing oviposition sites. If so, it is expected to collect more females over one kind of bait. However, no differences between female and male collected over baits were observed (see Table 2). Furthermore, only nine isofemales out of 69 (five collected over *H. uvarum* and four over *S. cerevisiae*) did not produced any progeny, so we have not much evidence of oviposition choice with this sample. Finally, further experiments evaluating differences in the choice behavior between females virgin and non virgin in the field as well as the relationship between yeast preference and components of biological fitness will be carried out. Moreover, the experimental design we used cannot rule out conspecific attraction (Lihoreau et al., 2016).

*H. uvarum* and *S. cerevisiae* have been already isolated from fruits and tree bark, respectively, in Amazonian and Atlantic Rainforests (Morais et al., 1995; Pimenta et al., 2009; Barbosa et al., 2016) as well as from crops of several *Drosophila* species (see Da Cunha, Shehata & De Oliveira, 1957; Morais et al., 1992; Morais, Pagnocca & Rosa, 2006; Batista et al., 2016). Species such as *D. paraguayensis* (see Batista et al., 2016) and *D. maculifrons* (see Da Cunha, Shehata & De Oliveira, 1957), which belong to *D. tripunctata* and *D. guarani* groups respectively, both of the *Drosophila* subgenus, had *S. cerevisiae* isolated from their crops. In contrast, *H. uvarum* group was the most prevalent yeast isolated from crops of *Sophophora* subgenus species such as: *D. willistoni* (see Da Cunha, Shehata & De Oliveira, 1957); *D. melanogaster* group (see Camargo & Phaff, 1957; Morais et al., 1995; Chandler, Eisen & Kopp, 2012); and *D. suzukii* (see Hamby et al., 2012).

Several evidences suggest the natural association between yeasts and *Drosophila* in the wild. Pimenta et al. (2009) states that *Drosophila* may be a major vector of yeasts in Atlantic Rainforest. Our data shows that species of *D. tripunctata* and *D. guarani* groups are preferentially attracted to *S. cerevisiae*, reflecting their natural association in the wild; while species of subgenus *Sophophora* such as *D. melanogaster*, which is preferentially attracted to baits with *H. uvarum* may be naturally associated with apiculate yeasts. Therefore, differences in dispersion and distribuition of the yeast species might be related to the variation observed for *Drosophila* species.

It is noteworthy that our results are consistent with those obtained more than half a century ago by researchers such as Da Cunha even if working with different objectives (see above) suggesting that the preferences among *Drosophila* species group found may be reflecting deep and stable relations with yeast species in natural forests in spite of all the environmental changes that have occurred. These results represent a first step to understand differences in feeding preferences among *Drosophila* species and their consequences for biological fitness. Naturally, additional studies characterizing yeast species associated with *Drosophila* species in natural remnants of Atlantic Rainforest, as well as the differences between male and female behavior, different physiological states, and on the molecular basis of *Drosophila* species olfactory system may further our understanding of the associations we now report.

##  Supplemental Information

10.7717/peerj.3063/supp-1 Data S1 Raw data List of drosophilids (females—sheet 1 and males—sheet 2) collected over uninoculated banana baits and banana baits with *Hanseniaspora uvarum* and *Saccharomyces cerevisiae*.

## Additional Information and Declarations

### Field Study Permissions

The following information was supplied relating to field study approvals (i.e., approving body and any reference numbers): Ministério do Meio Ambiente—MMA Instituto Brasileiro do Meio Ambiente e dos Recursos Naturais Renováveis—IBAMA Instituto Chico Mendes de Conservação da Biodiversidade—ICMBio Licença permanente para coleta de material zoológico: Número: #17238-1.

### Data Availability

The following information was supplied regarding data availability: The raw data has been supplied as Data S1.
